# Orlistat exerts anti-obesity and anti-tumorigenic effects in a transgenic mouse model of endometrial cancer

**DOI:** 10.3389/fonc.2023.1219923

**Published:** 2023-08-04

**Authors:** Guangxu Xu, Ziyi Zhao, Weiya Z. Wysham, Dario R. Roque, Ziwei Fang, Wenchuan Sun, Yajie Yin, Boer Deng, Xiaochang Shen, Chunxiao Zhou, Victoria Bae-Jump

**Affiliations:** ^1^ Department of Gynecology, Fengxian Hospital, Southern Medical University, Shanghai, China; ^2^ Division of Gynecologic Oncology, University of North Carolina at Chapel Hill, Chapel Hill, NC, United States; ^3^ Department of Gynecologic Oncology, Beijing Obstetrics and Gynecology Hospital, Beijing Maternal and Child Health Care Hospital, Capital Medical University, Beijing, China; ^4^ Division of Gynecologic Oncology, Legacy Medical Group, Portland, OR, United States; ^5^ Division of Gynecologic Oncology, Northwestern University Feinberg School of Medicine, Chicago, IL, United States; ^6^ Lineberger Comprehensive Cancer Center, University of North Carolina at Chapel Hill, Chapel Hill, NC, United States

**Keywords:** orlistat, obesity, endometrial cancer, apoptosis, dietary intervention

## Abstract

**Introduction:**

Among all cancers, endometrial cancer is most strongly associated with obesity, with more than 65% of endometrial cancers attributable to obesity and being overweight. Fatty acid synthase (FAS), a key lipogenic enzyme, is expressed in endometrial cancer tumors and is associated with a worse prognosis for this disease. Orlistat, an FAS inhibitor, is an FDA-approved weight loss medication that has demonstrated anti-tumor activity in a variety of preclinical cancer models.

**Methods:**

In this study, the *Lkb1^fl/fl^p53^fl/fl^* mouse model of endometroid endometrial cancer was exposed to three diet interventions, including a high fat diet (obese), a low fat diet (lean) and switch from a high fat to a low fat diet, and then exposed to orlistat or placebo.

**Results:**

The mice fed a high-fat diet had significantly increased body weight and tumor weight compared to mice fed a low-fat diet. Switching from a high-fat diet to a low fat diet led to a reduction in mouse weight and suppressed tumor growth, as compared to both the high fat diet and low fat diet groups. Orlistat effectively decreased body weight in obese mice and inhibited tumor growth in obese, lean, and the high fat diet switch to low fat diet mouse groups through induction of apoptosis. Orlistat also showed anti-proliferative activity in nine of 11 primary cultures of human endometrial cancer.

**Discussion:**

Our findings provide strong evidence that dietary intervention and orlistat have anti-tumor activity *in vivo* and supports further investigation of orlistat in combination with dietary interventions for the prevention and treatment of endometrial cancer.

## Introduction

Endometrial cancer (EC) is the most common malignancy of the female reproductive tract, with an estimated 66,200 incident cases and 13,030 deaths in the United States in 2023 (1). Among the risk factors related to EC, obesity is most strongly associated with carcinogenesis and progression of EC, as compared to any other cancer type. The morbidity and mortality from EC have continued to increase in recent decades as compared with many other cancer types, due in part to an increasing prevalence of obesity (2, 3). The five-year survival rate for patients with early-stage EC exceeds 85-90%. However, the chance of five-year survival is low in EC patients with recurrent or advanced disease, with a 15-25% chance of survival in stage IV patients (4, 5). Because of a lack of effective treatment strategies for advanced and recurrent disease, there is a need to identify and develop a new generation of therapeutic agents and treatment options for this disease.

More than 50% of EC patients in the United States are overweight or obese, and weight loss has been shown to be efficient in reducing EC risk and overall mortality (6, 7). Mounting evidence has demonstrated that fatty acid synthase (FAS) is critical in the metabolic reprogramming in EC and is a target for the development of anti-obesity and anti-cancer drugs (8–10). The expression of FAS increases gradually from endometrial hyperplasia to carcinoma, and inhibition of FAS activity by siRNA downregulates E2-stimulated ERα expression and inhibits cell viability in EC cells, indicating that FAS is associated with carcinogenesis of EC and may be a potential therapeutic target for EC (11, 12). Orlistat, an irreversible inhibitor of FAS, is an FDA-approved anti-obesity agent that is widely available worldwide. Numerous research studies have confirmed that orlistat exhibits anti-tumorigenic activities against various types of solid and hematological malignancies, including prostate, leukemia, breast, ovarian, colon, gastric, melanoma, and oral cancers through induction of cell cycle arrest and apoptosis in preclinical models, with a minor inhibitory effect on normal cells (13–16). Our previous study found that orlistat inhibited cell viability through inhibition of fatty acid metabolism, induction of cell cycle G1 arrest, activation of AMPK, and inhibition of the mTOR pathway in EC cell lines (17).

Given that obesity promotes EC carcinogenesis and progression, and orlistat effectively inhibits EC cell growth *in vitro*, we evaluated the anti-tumorigenic efficacy of orlistat in primary cultures of human endometrial cancer as well as in a genetically engineered mouse model of endometrioid endometrial cancer under obese and lean conditions. Our goal was to provide pre-clinical data for the potential use of orlistat in the treatment and prevention of EC.

## Materials and methods

### Reagents

Orlistat was purchased from Cayman Chemical Company (Cat #: 10005426, Ann Arbor, MI) and solubilized in 33% ethanol and 66% PEG400 for animal use. The antibodies used in the experiments were from Cell Signaling (Beverly, MA). RIPA buffer, MTT, and enhanced chemiluminescence western blotting detection reagents were purchased from Thermo Fisher Scientific (Waltham, MA). A cleaved caspase-3 assay was procured from Novus Biologicals (Centennial CO). Glucose assays were purchased from AAT Bioquest (Sunnyvale, CA). Free fatty acid and triglyceride quantification assays were procured from BioVision (Waltham, MA).

### Biochemical analysis

The mouse LUMINEX assays (Milliplex Map, Millipore CA) were used to detect the productions of serum leptin, insulin, TNF-α, and IL-6 according to the manufacturer’s protocols in the University of North Carolina at Chapel Hill (UNC-CH) animal core facility. Data was collected and analyzed using the Luminex-200 system (Austin, TX).

### Collection of EC tissue samples and primary endometrioid EC cell cultures

Eleven tumor specimens were collected from EC patients undergoing a hysterectomy at UNC-CH Hospital, after informed consent was obtained. The protocol was reviewed and approved by the Institutional Review Board (IRB) of UNC-CH. The fresh tissues were gently washed with PBS and then minced by scissors and scalpels in DMEM/F12 medium containing 10% fetal bovine serum (FBS). 1.5 × 10^4^ primary culture cells/well were seeded into 96-well plates, and 5-6 × 10^5^ cells/well were cultured in 6-well plates. After incubating overnight, the cells were treated with varying concentrations of orlistat for 24 hours or 72 hours. Cell proliferation was measured by MTT assay after 72 hours of treatment. For western blotting, cells were lysed by RIPA assay after 24 hours of treatment.

### MTT assay

Primary EC cells were plated at a density of 1.5 × 10^4^ cells/well in 96-well plates and allowed to adhere overnight. The cells were treated with vehicle or different concentrations of orlistat for 72 hours. After treatment, 100 µl of MTT (5 mg/ml in DMEM/F12 medium) were added to each well and incubated for two hours at 37°C. The medium was removed, and 100 µl of DMSO was added to each well. Absorbance was measured at 570 nm using a Tecan plate reader (Morrisville, NC). The value of IC50 was calculated by IC50 Calculator (ATT Bioquest).

### Cleaved caspase-3 assay

The primary culture cells were plated in 6-well plates at a concentration of 8 x 10^5^ cells/well. After 24 hours of growing, cells were exposed to different concentrations of orlistat for 14 hours. 150-180 µl of 1X caspase lysis buffer was added into each well, and protein concentrations were determined with BCA assay (Thermo Fisher Scientific). In a new black 96-well plate, the reaction buffer containing caspase-3 substrate (AAT Bioquest) was mixed with lysis buffer for 20 minutes at 37°C. The fluorescence intensity (Ex/Em=400/505) for cleaved caspase 3 was determined using a Tecan microplate reader.

### Diet interventions and orlistat treatment in *Lkb1^fl/fl^p53^fl/fl^* mice

The *Lkb1^fl/fl^p53^fl/fl^* mouse model is an endometrioid EC mouse model that was created by our team (18). All animal procedures were approved by the Institutional Animal Care & Use Committee (IACUC) of UNC-CH (protocol # 21-229). The *Lkb1^fl/fl^p53^fl/fl^* mice were housed in a 12-hour light/dark cycle with a temperature of 22 ± 2°C. The mice were given free access to food and water. Mice were fed a high fat diet (HFD, 60 kcal% fat; Research Diets, New Brunswick, NJ) to induce obesity, or a low fat diet (LFD, 10 kcal% Fat; Research Diets), at three weeks of age. At six to eight weeks of age, *LKb1^fl/fl^p53^fl/fl^* mice were injected with one-sided intrauterine AdCre (University of Iowa Transfer Vector Core, a titer of 10^11^-10^12^) to induce EC. After eight weeks of injection, mice were randomly divided into six groups (n=20/group): HFD+placebo, HFD+orlistat, LFD+placebo, LFD+orlistat, HFD-LFD+placebo (i.e., switching mice from an HFD to LFD), and HFD-LFD+orlistat. Orlistat (60 mg/kg or 200 mg/kg, daily) was administered to mice via oral gavage for four weeks. Placebo treatment consisted of orally administering to mice an equivalent solution of dissolved orlistat. Body weight and blood glucose were measured twice a week. At the end of the experiments, blood samples were obtained by retro-orbital vein collection under anesthesia, and the serum was separated by centrifuge for 20 minutes and stored at 80°C until assayed. Tumor tissues, livers, and visceral adipose tissues, including gonadal fat pads, were weighted, collected, and stored in liquid nitrogen. Pieces of gonadal adipose pads, livers, and tumor tissues were fixed in 10% formaldehyde for subsequent histological and immunohistochemical analysis.

### Histological analysis

The tissues were embedded in paraffin after being fixed in 10% formaldehyde according to standard procedures in the UNC-CH animal core facility. Paraffin-embedded tissue blocks, including tumor, liver, and adipose tissues, were cut into 5-micron-thick sections. Sections were stained with hematoxylin and eosin (H&E) for morphological observations under an optical microscope (Olympus CX41, Olympus Optical Co., Tokyo, Japan), and the images were scanned into the Motic scanner (Meyer Instruments, Houston, TX).

### Immunohistochemistry

Endometrial tumor slides (4 µm) from LKB1^fl/fl^ p53^fl/fl^ mice were first incubated with protein block solution (Dako) for one hour and then added with the primary antibodies overnight in a cold room. The slides were then washed with TBS-T and incubated with appropriate secondary antibodies at room temperature for one hour. After removing the secondary antibody, the specific staining was visualized using the Signal Stain Boost Immunohistochemical Detection Reagent (Cell Signaling Technology, Danvers, MA), according to the manufacturer’s instructions. Individual slides were scanned using the Motic scanner, and digital images were analyzed for target protein expression using ImagePro software (Vista, CA).

### Western immunoblotting

Following treatment of primary culture cells with different concentrations of orlistat overnight, total proteins were extracted from both cell lines using Tissue-PE LB buffer (G-Biosciences, St. Louis, MO). A BCA protein assay kit (Thermo Fisher Scientific) was used to determine the concentration of protein. Equal amounts of protein were separated by gel electrophoresis and transferred onto a PVDF membrane. The membranes were blocked with 5% non-fat dry milk and then incubated with 1:1000 dilutions of Bcl-xL, MMP-9, and VEGF antibodies overnight at 4°C. The membrane was washed with TBS-T and incubated with the appropriate secondary antibody for one hour at room temperature. Immunoblots were developed using an enhanced chemiluminescence detection buffer, and bands were visualized with the Bio-Rad Imaging System (Hercules, CA). After development, the membranes were washed and re-probed using antibodies against α-tubulin. Each experiment was repeated at least twice to assess consistency.

### Serum VEGF and triglyceride assays

The VEGF productions of mice serum and serum triglyceride levels after treatment with Orlistat were detected using an R&D VEGF ELISA Kit (Minneapolis, MN) and a BioVision Triglyceride Quantification Kit, respectively, according to the manufacturer’s directions. Each sample was measured in triplicate. The plates were read at 570 nm for VEGF and triglyceride measurements using a Tecan plate reader.

### EchoMRI scan

Measurements of body composition in obese and lean mice groups, including lean body mass, fat mass, free water content, and total water content, was performed using the Whole-Body Composition Analyzer by quantitative magnetic resonance imaging EchoMRI-100 in a UNC-CH animal facility, according to the manufacturer’s instructions.

### Statistical analysis

Statistical analysis was performed using GraphPad Prism 8 (La Jolla, CA). All data are reported as mean ± SD from three independent assays. Both Student’s t-test and two-way ANOVA test were used in this study. The values of p < 0.05 and p < 0.01 are considered statistically significant (*) and very significant (**), respectively.

## Results

### Effect of orlistat on body weight in mice fed a HFD, LFD and a HFD switched to a LFD

**Figure 1:**
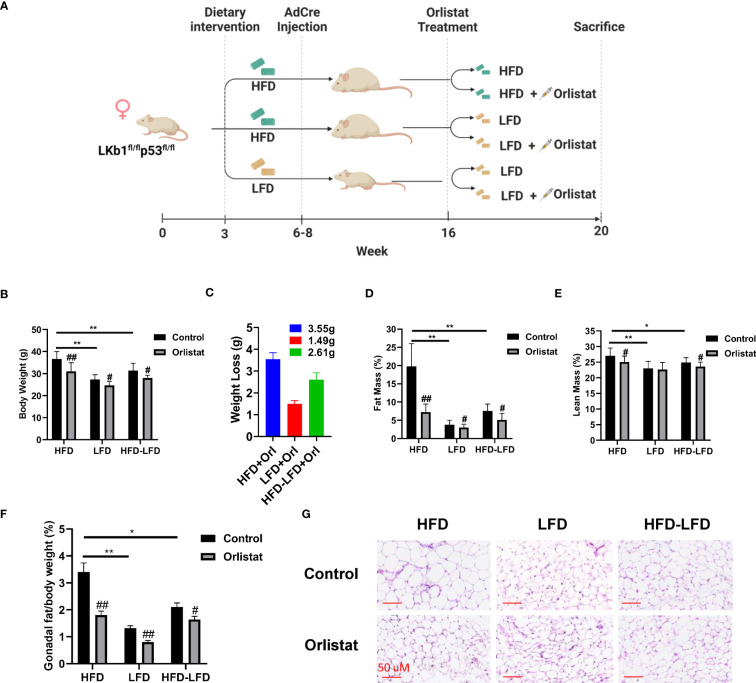
Effect of orlistat on body weight in mice fed a HFD or LFD. The *Lkb1^fl/fl^p53^fl/fl^* mice fed either a HFD or LFD were divided into six diet/drug intervention groups, and the experimental strategy is shown in **(A)**. The body weights of mice from control and orlistat treatment groups were measured at the end of treatment **(B)**. The average body weight loss in orlistat-treated groups **(C)**. Measurement of fat and lean mass of mice from control and orlistat-treated groups using EchoMRI **(D, E)**. Orlistat reduced the ratio of gonadal fat/body weight in each group **(F)**. H&E staining showed that orlistat decreased the size of gonadal adipocytes in HFD, LFD, and HFD-LFD mice **(G)**. *p< 0.05, **p< 0.01. ^#^Indicates comparison with corresponding control, ^#^p< 0.05, ^##^p<0.01.

To investigate the effect of orlistat on body weight under different obese (HFD-fed) and lean (LFD-ed) conditions, *Lkb1^fl/fl^p53^fl/fl^* mice were randomly distributed into six groups (n=20): HFD+placebo, HFD+orlistat, LFD+placebo, LFD+orlistat, HFD switched to LFD (HFD-LFD)+placebo, and HFD-LFD+orlistat, with orlistat (60 mg/kg, oral garage) administered for four weeks (**Figure 1A**). Consistent with our previous study (18), HFD significantly promoted body weight gain by 34.53% in HFD-fed mice when compared to LFD-fed mice (**Figure 1B**). Treatment of the mice with orlistat for four weeks as compared to placebo reduced body weight in each of the diet intervention groups. In addition, switching from HFD to LFD for four weeks resulted in a significant 10.72% decrease in body weight, producing similar body weight effects as orlistat in the HFD group. Orlistat induced weight loss in the HFD, LFD, and HFD-LFD groups by 3.55, 1.49, and 2.61 g, respectively, compared to pre- and post-treatment body weight (**Figure 1C**). EchoMRI results showed that at the end of the treatment, the HFD group had nearly five times as much total body fat as the LFD control group. Orlistat treatment significantly decreased fat mass in the HFD, LFD, and HFD-LFD groups, as compared to the respective placebo control mice. In addition, orlistat reduced lean mass in the HFD and HFD-LFD groups but not in the LFD group (**Figures 1D, E**). Since different visceral fat pad weights reflect distensibility under obese conditions, and gonadal fat pad weights increase primarily during the initial phase of weight gain (19), gonadal fat weight was collected and measured, and the percentage of gonadal fat weight relative to body weight was calculated in each group. Consistent with the EchoMRI results, orlistat reduced the ratios of gonadal fat/body weight in each diet intervention group, with the strongest effects in HFD mice (**Figure 1F**). Furthermore, H&E results showed that the gonadal adipocytes were larger in the HFD group, and orlistat or LFD significantly reduced the gonadal adipocyte size, as compared to HFD control mice (**Figure 1G**). These results suggest that orlistat reduces body weight primarily by reducing fat mass, especially in the obese state.

### Effect of orlistat on hepatic fat deposition and serum biochemical parameters in HFD, LFD and HFD-LFD mice

**Figure 2:**
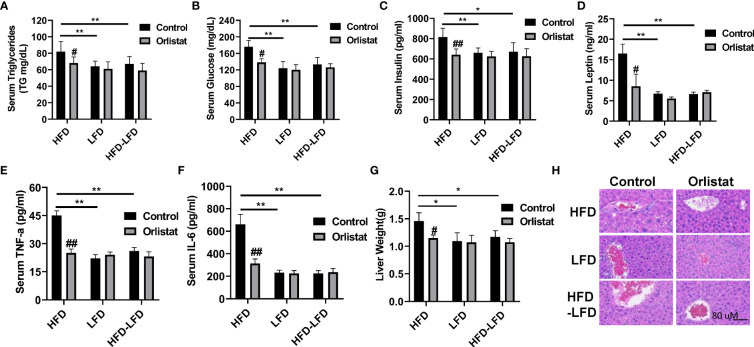
Effect of orlistat on hepatic fat deposition and serum biochemical parameters in HFD, LFD HFD-LFD mice. Serum levels of TG, glucose, insulin, and leptin were significantly elevated in HFD mice compared with LFD and HFD-LFD groups, and treatment of mice with orlistat for four weeks reduced serum TG, glucose, insulin, and leptin productions in the HFD group **(A–D)**. The inflammatory factors IL-6 and TNF-α were elevated in HFD mice, and orlistat treatment significantly decreased serum IL-6 and TNF-α levels in these mice **(E, F)**. The liver weight was significantly increased in mice fed a HFD, and treatment with orlistat for 4 weeks effectively reduced liver weight **(G)**. H&E staining result showed that HFD induced balloon-like structures in liver tissues, and orlistat alleviated those pathologic changes in the livers **(H)**. *p<0.05, **p<0.01. ^#^compared with HFD-fed mice. ^##^p<0.01.

Given that orlistat markedly reduced body weight in the three diet intervention groups (HFD, LFD, HFD-LFD), particularly in the HFD group, serum triglycerides (TGs), insulin, and leptin were examined by ELISA assays. Mice in the HFD group significantly increased serum TG production compared to the LFD and HFD-LFD mice, while orlistat treatment effectively reduced the TG levels induced by HFD (**Figure 2A**). Similar effects were observed for serum glucose and insulin, with HFD increasing serum glucose, insulin, and leptin concentrations in HFD mice but not in the LFD and HFD-LFD groups. Orlistat treatment of HFD mice showed significant improvements in serum glucose, insulin, and leptin, whereas orlistat did not affect changes in serum glucose, insulin, and leptin in the LFD and HFD-LFD groups (**Figures 2B–D**). Since obesity triggers a chronic low-grade inflammatory response, blood samples from all three dietary intervention groups were analyzed for inflammatory factors IL-6 and TNF-α. The serum levels of IL-6 and TNF-α in HFD-fed mice were higher than those in LFD-fed and HFD-LFD mice, but there were no differences in IL-6 and TNF-α between the LFD and HFD-LFD groups. In the HFD, LFD, and HFD-LFD groups, four weeks of treatment with orlistat only significantly improved serum levels of IL6 and TNF-α in the HFD group, but not in the LFD and HFD-LFD groups. (**Figures 2E, F**).

Since long-term HFD significantly increases liver weight compared to mice on a normal diet (20), the effect of HFD on hepatic fat deposition was investigated. As expected, liver weight was significantly increased in the HFD group compared to the LFD and HFD-LFD groups (**Figure 2G**). Administration of orlistat or switching from a HFD to LFD resulted in a significant reduction in liver weight of 21.23% and 19.86%, respectively. H&E staining of liver sections confirmed that mice in the HFD group exhibited distinct balloon-like structures compared with the LFD and HFD-LFD groups, and treatment with orlistat or switching from a HFD to an LFD for four weeks alleviated these pathologic changes in the liver tissues (**Figure 2H**).

### Effect of orlistat on tumor growth in HFD, LFD and HFD-LFD mice

**Figure 3:**
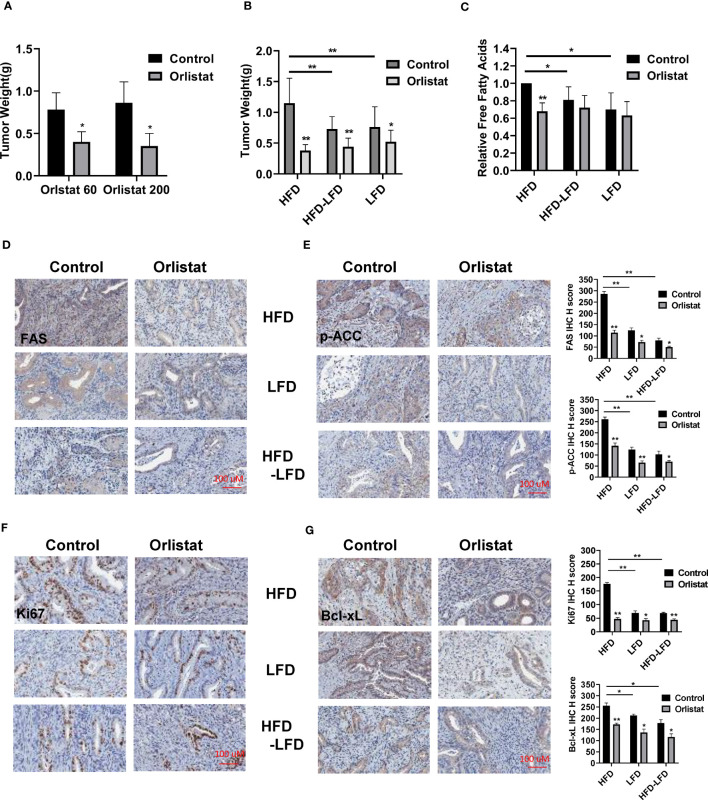
Orlistat inhibited tumor growth in HFD, LFD and HFD-LFD mice. HFD, LFD and HFD-LFD mice were treated with orlistat at 60 mg/kg for four weeks. Orlistat at 60 and 200 mg/kg effectively inhibited EC tumor growth **(A)**. HFD significantly promoted tumor growth compared to an LFD and HFD-LFD, and orlistat treatment (60 mg/kg) for 4 weeks inhibited tumor growth in HFD, LFD, and HFD-LFD mice **(B)**. The measurements of free fatty acid in tumor tissues in each group **(C)**. The EC tumor tissues in each group were stained with FAS, p-ACC, Ki67, and Bcl-xL. The results showed orlistat significantly reduced the expression of FAS, p-ACC, Ki67, and Bcl-xL in HFD, LFD, and HFD-LFD mice **(D–G)**. *p<0.05, **p<0.01. ^#^Indicates comparison with corresponding control, ^#^p< 0.05, ^##^p<0.01.

Since orlistat has been shown to inhibit tumor growth over a wide range of concentrations in different mouse models, we initially selected two doses of orlistat (60 mg/kg and 200 mg/kg, daily) to treat *Lkb1^fl/fl^p53^fl/fl^* mice for four weeks. At the end of treatment, orlistat showed similar inhibition of tumor growth in both groups compared to vehicle controls (**Figure 3A**). Next, to determine the effectiveness of orlistat on tumor growth in *Lkb1^fl/fl^p53^fl/fl^* mice under obese and lean conditions, the mice in the HFD, LFD, and HFD-LFD groups were treated with orlistat (60mg/kg) for four weeks. Consistent with our previous studies (4, 18, 21), a HFD significantly increased tumor weight by 36.34% compared to LFD-fed mice (1.15 g vs 0.76 g) and 33.41% compared to HFD-LFD-fed mice (1.15 g vs 0.73 g), respectively (**Figure 3B**). Orlistat effectively inhibited tumor growth by 67.06%, 40.36%, and 32.62% in the HFD, HFD-LFD, and LFD groups, respectively, compared with placebo-treated mice in each group. The four-week switch from HFD-LFD also effectively reduced tumor weight compared to the HFD group, suggesting that energy restriction is an effective strategy to inhibit tumor growth in EC.

Given that orlistat is a potent inhibitor of FAS, free fatty acids were measured by ELISA assay in EC tumors of *Lkb1^fl/fl^p53^fl/fl^* mice. The results showed that orlistat significantly decreased free fatty acids in HFD mice as compared to control mice, but not in the HFD-LFD and LFD groups (**Figure 3C**). Similarly, compared with HFD mice, switching from HFD-LFD for four weeks reduced free fatty acids in EC tumors. More importantly, IHC staining results showed that orlistat treatment significantly reduced the expression of fatty acid synthase (FAS) and phos-ACC in the HFD, HFD-LFD, and LFD groups (**Figures 3D, E**). Positive staining for Ki67 and Bcl-xL in the HFD, HFD-LFD and LFD mice treated with orlistat also decreased significantly, with the most pronounced effect in HFD mice (**Figures 3F, G**).

### Effect of orlistat on angiogenesis in HFD, LFD and HFD-LFD mice

**Figure 4:**
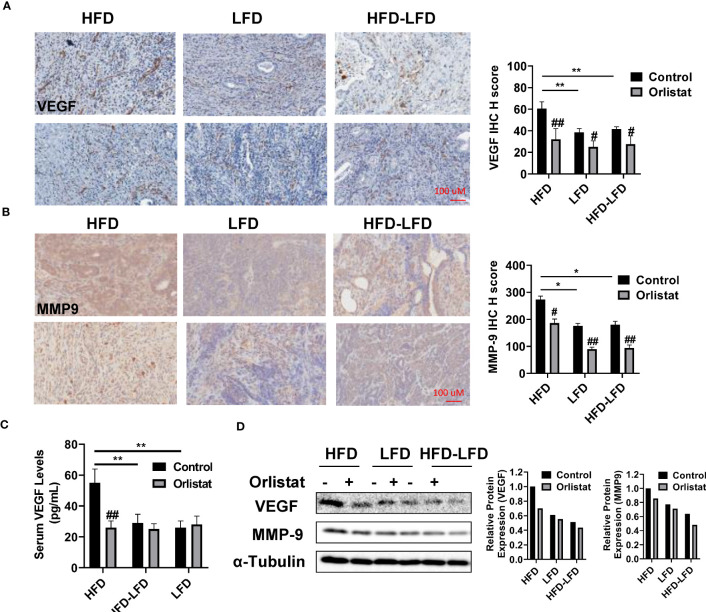
Orlistat reduced angiogenesis in obese and lean mice. The expression of VEGF and MMP-9 were measured by IHC staining in EC tumors of *Lkb1^fl/fl^p53^fl/fl^* mice. Orlistat downregulated the expression of VEGF in the HFD and HFD-LFD groups, as well as MMP-9 in all three groups (HFD, LFD and HFD-LFD) **(A, B)**. Orlistat treatment reduced serum VEGF levels in HFD mice **(C)**. Western blotting results demonstrated that orlistat treatment for four weeks also reduced the expression of VEGF and MMP-9 in the EC tumors **(D)**. *p<0.05, **p<0.01. ^#^Indicates comparison with corresponding control, ^#^p< 0.05, ^##^p<0.01.

Since orlistat exhibited anti-invasive capacity in preclinical models (15, 16), the expression of VEGF and MMP9 were measured by IHC staining in the EC tumors of *Lkb1^fl/fl^p53^fl/fl^* mice. HFD promoted the expression of VEGF and MMP-9 compared to HFD-LFD and LFD mice, while orlistat significantly reduced the expression of VEGF in the HFD and HFD-LFD groups, as well as the expression of MMP-9 in the three diet intervention groups (**Figures 4A, B**). Serum VEGF levels also rose in HFD mice, with an average peak level of 55.46 pg/ml, compared to 32.13 pg/ml in HFD-LFD mice and 26.75 pg/ml in LFD mice. Orlistat treatment effectively reduced the levels of VEGF in the HFD group but did not show the same effect on serum VEGF expression in the HFD-LFD and LFD groups (**Figure 4C**). Western blotting results from EC tumors demonstrated that orlistat reduced the expression of VEGF and MMP-9 in the HFD, LFD, and HFD-LFD mice (**Figure 4D**).

### Effect of orlistat on cell proliferation in primary cultures of human EC

**Figure 5:**
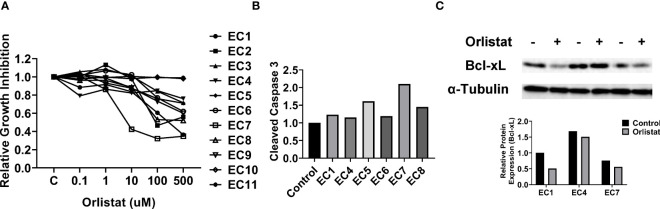
Orlistat inhibited cell proliferation in primary cultures of human EC. The effect of orlistat on cell proliferation was detected by MTT assay in 11 primary cultures of human endometrioid ECs **(A)**. Changes of cleaved caspase-3 were measured by ELISA assay in EC1, EC4, EC5, EC6, EC7, and EC8 cells **(B)**. The expression of Bcl-xL in EC1, EC4, and EC7 samples was assessed by western blotting after 24 hours of treatment with orlistat **(C)**.

As primary cell cultures of human EC may be better predictors of anti-tumorigenic activity of cytotoxic agents than cancer cell lines (22, 23), 11 primary cell cultures from patients with endometrioid endometrial adenocarcinoma were used to detect the effect of orlistat on cell viability (**Table 1**). The primary culture cells were treated with 0.1, 1, 10, 100 and 500 µM orlistat for 72 hours. Cell proliferation was assessed by MTT assay. The results showed that nine of 11 primary culture cells had differential inhibitory responses to orlistat treatment, with two cases showing IC50 values of 25.4 µM and 146.4 µM (**Figure 5A**). Next, we performed a cleaved caspase-3 assay on primary cell cultures from six of the nine cases that responded to orlistat. Orlistat increased cleaved caspase-3 activity in all six primary cultures of EC cells. 100 µM orlistat effectively increased cleaved caspase-3 activity by 2.1-fold in primary EC7 cells, which were the most sensitive to orlistat (**Figure 5B**). Additionally, western blotting results showed that orlistat significantly decreased the expression of Bcl-xL in these EC primary cultures after 24 hours of treatment with 50 µM orlistat (**Figure 5C**).

**Table 1:** Clinical and pathological characteristics of 11 patients with endometrial carcinoma.

Case	Age	Race	Stage	Tumor size(cm)	Histology	HER2
EC1	54	Hispanic (White)	IA	3.5	Endometrioid, grade 2	Negative
EC2	73	White	IA	4.5	Endometrioid, grade 1	Negative
EC3	46	Hispanic (White)	IA	2.2	Endometrioid, grade 1	N/A
EC4	46	Black	IA	3.5	Endometrioid, grade 3	N/A
EC5	63	Black	IA	4.5	Endometrioid, grade 1	N/A
EC6	43	Black	IIIC1	0.5	Endometrioid, grade 1	N/A
EC7	80	White	IB	5.3	Endometrioid, grade 2	N/A
EC8	40	White	IA	3.7	Endometrioid, grade 1	N/A
EC9	67	White	IA	4.8	Endometrioid, grade 2	N/A
EC10	74	White	IB	6	Endometrioid, grade 1	N/A
EC11	54	Hispanic (White)	IA	3.3	Endometrioid, grade 1	N/A

## Discussion

We previously reported that orlistat exhibited anti-proliferative activity against EC cells *in vitro* by inducing cell cycle G1 arrest and cellular stress and inhibiting fatty acid metabolism and the mTOR pathway, indicating that orlistat deserves further anti-tumor studies *in vivo* for EC (17). Considering that more than 50% of patients with EC are overweight or obese, and the expression of FAS is closely associated with the progression of EC, weight loss or targeting FAS can be proposed as a novel approach to treat or prevent the development of EC or to improve survival by reducing obesity-related comorbidities (17, 24–26). Orlistat is an FAS-specific inhibitor and FDA-approved weight loss drug that has demonstrated anti-tumorigenic activity against multiple cancers in pre-clinical models (13, 26). In the present study, we investigated the effect of orlistat on body weight, lipid metabolism, and tumor growth in a transgenic mouse model of endometrioid EC under obese and lean conditions. Our results confirmed that systemic treatment with orlistat significantly reduced the body weight in HFD, LFD, and HFD-LFD mice by impairing lipogenic potential and reducing fat mass, with the greatest effect on body weight in HFD-fed mice. More importantly, orlistat effectively attenuated tumor weight by inhibiting FAS and inducing apoptosis in all three diet intervention mouse groups (HFD, LFD, HFD-LFD), and decreased angiogenesis by reducing the expression of MMP-9 and VEGF of EC tumors in HFD and HFD-LFD mice. Primary culture data from human endometrioid ECs also supported the anti-proliferative potential of orlistat for this disease.

Obesity-associated chronic metabolic dysfunctions are considered to be major contributing factors for the carcinogenesis and progression of EC through various mechanisms, including insulin resistance, increased amount of visceral adipose tissue and serum adipokines, activated aromatase signaling pathways, and an elevated chronic inflammatory status (27–29). These metabolic dysfunctions can be controlled or reversed by long-term pharmacological agents, dietary or surgical interventions, or by physical activity, potentially reducing the risk of EC and increasing survival in patients with EC (7, 25, 30). Recent systematic reviews showed that intentional weight loss, including diet and exercise interventions and surgical weight loss interventions, improved biomarkers of EC, including circulating hormones and tissue markers, and lowered the risk of developing EC (31, 32). We used three dietary interventions in combination with orlistat to evaluate the effect of obesity and obesity-related factors on tumor growth in the *Lkb1^fl/fl^ p53^fl/fl^* mouse model of endometroid EC. Consistent with our previous studies (18, 33, 34), HFD significantly increased body weight and promoted EC tumor growth, whereas HFD-LFD effectively reduced tumor weight compared with HFD mice. Although dietary control produced a pronounced effect on tumor growth in diet-induced obese mice, orlistat exhibited potent anti-tumor activity in the HFD, LFD, and HFD-LFD mice that was most pronounced in the HFD group. HFD leads to increases in lipogenesis, resulting in the upregulation of serum lipid and glucose profiles and the elevation of pro-inflammatory factor, including glucose, TNF-a, IL-8, TG, and cholesterol, which are implicated in increased proliferation of EC cells. Not surprisingly, reduction of fat accumulation by either switching from a HFD to a LFD or orlistat treatment caused normalization of body weight, reduced the levels of serum glucose, TG, proinflammatory factors, and decreased the expression of FAS and p-ACC in tumor tissues. However, this did not seem to be the mechanism of action of orlistat in the LFD mice. Thus, the inhibitory effect of orlistat on tumor progression in these HFD-fed, HFD-LFD-fed and LFD-fed mice may have different mechanisms, particularly in the HFD mice.

Inhibition of FAS results in the accumulation of toxic intermediary metabolite malonyl-CoA, which impairs membrane synthesis and phospholipid function and exhibits cytotoxic effects in cancer cells. The anti-tumor properties of orlistat involve multiple mechanisms, such as causing cell cycle G1 arrest, inducing cellular stress, and initiating apoptosis through multiple cellular signaling pathways in cancer cells (26, 35, 36). Our previous study showed that orlistat inhibited cell proliferation through inhibition of fatty acid metabolism, induction of cell cycle G1 arrest, and inhibition of the mTOR pathway, but it did not increase annexin V expression or caspase-3 activity in established human EC cell lines (17). The results of the current study showed that orlistat reduced the expression of Bcl-xL in tumor tissues of HFD, LFD, and HFD-LFD mice, suggesting that orlistat can induce apoptosis *in vivo* after four weeks of treatment. Given that orlistat demonstrated strong inhibitory effects on lipid metabolism and inflammatory factors in HFD but not LFD mice, we postulate that apoptotic pathways may be the primary underlying biological mechanisms through which orlistat reduces tumor growth in LFD mice.

Expansion of adipose tissue during the development of obesity effectively activates the cascade of angiogenesis and enhances the expression of VEGF (37). Inhibition of FAS impairs physiological and pathological angiogenesis in endothelial cells, ultimately resulting in reduced vessel sprouting and neovascularization (38). A strong positive correlation between the expression of FAS and VEGF was found in human colorectal cancer, and cancer cell-associated FAS regulated tumor vasculature by altering the distribution of secreted angiogenic factors (39, 40). Targeting FAS by shRNAs reduced tumor growth and microvessel density in human gliomas (41). As an inhibitor of FAS, orlistat has been reported to have anti-angiogenic activity, including regulating the production of total VEGFA in cancer cells, reducing the formation of capillary-like structures, and decreasing the number of metastatic cervical lymph nodes in BALB/c nude mice (15, 16, 42). Concomitantly, we found that orlistat treatment or switching from a HFD to a LFD significantly inhibited the expression of VEGF and MMP-9 in tumor tissues of HFD and HFD-LFD mice and decreased serum VEGF levels in HFD mice, indicating that inhibition of FAS by orlistat may restrain the invasive capacity of EC. Although angiogenic activity is primarily controlled by the VEGF/VEGF-receptor system in adipose and tumor tissues, the biological mechanism by which orlistat reduces angiogenic capacity needs to be elucidated in future studies.

In conclusion, our results showed that HFD-induced obesity promoted more aggressive tumor growth, whereas the dietary intervention of switching from a HFD to a LFD significantly reduced body weight, improved lipid metabolism, inhibited inflammatory factors production, and reduced tumor growth in the *Lkb1^fl/fl^ p53^fl/fl^* mouse model of EC. Orlistat treatment also reversed HFD-driven lipogenesis and impaired angiogenesis and tumor growth through the inhibition of FAS and induction of apoptosis under obese and lean conditions. This study provides a mechanistic link between obesity, dietary interventions, and orlistat in modulating tumor growth of EC, and it supports further investigation of orlistat alone or in combination with other dietary interventions for the prevention and treatment of EC.

## Data availability statement

The raw data supporting the conclusions of this article will be made available by the authors, without undue reservation.

## Ethics statement

The protocol for collecting human EC tissues was reviewed and approved by the Institutional Review Board (IRB) of UNC-CH. The patients/participants provided their written informed consent to participate in this study. The animal study was approved by the Institutional Animal Care & Use Committee (IACUC) of the UNC-CH. The protocol for collecting human EC tissues was reviewed and approved by the Institutional Review Board (IRB) of UNC-CH.

## Author contributions

Conceptualization: VB-J and CZ. Methodology and experimental design: GX, ZZ, WW, DR, ZF, WS, YY and CZ. Data collection and interpretation: GX, ZZ, ZF, WS, BD, XS, YY and CZ. Drafted manuscript: CZ and VB-J. All authors contributed to the article and approved the submitted version.
